# Involvements of PCD and changes in gene expression profile during self-pruning of spring shoots in sweet orange (*Citrus sinensis*)

**DOI:** 10.1186/1471-2164-15-892

**Published:** 2014-10-13

**Authors:** Jin-Zhi Zhang, Kun Zhao, Xiao-Yan Ai, Chun-Gen Hu

**Affiliations:** Key Laboratory of Horticultural Plant Biology (Ministry of Education), College of Horticulture and Forestry Science, Huazhong Agricultural University, Wuhan, 430070 China

**Keywords:** Abscission zone, Citrus, Microarray, Programmed cell death, Self-pruning, Shoot tips

## Abstract

**Background:**

Citrus shoot tips abscise at an anatomically distinct abscission zone (AZ) that separates the top part of the shoots into basal and apical portions (citrus self-pruning). Cell separation occurs only at the AZ, which suggests its cells have distinctive molecular regulation. Although several studies have looked into the morphological aspects of self-pruning process, the underlying molecular mechanisms remain unknown.

**Results:**

In this study, the hallmarks of programmed cell death (PCD) were identified by TUNEL experiments, transmission electron microscopy (TEM) and histochemical staining for reactive oxygen species (ROS) during self-pruning of the spring shoots in sweet orange. Our results indicated that PCD occurred systematically and progressively and may play an important role in the control of self-pruning of citrus. Microarray analysis was used to examine transcriptome changes at three stages of self-pruning, and 1,378 differentially expressed genes were identified. Some genes were related to PCD, while others were associated with cell wall biosynthesis or metabolism. These results strongly suggest that abscission layers activate both catabolic and anabolic wall modification pathways during the self-pruning process. In addition, a strong correlation was observed between self-pruning and the expression of hormone-related genes. Self-pruning plays an important role in citrus floral bud initiation. Therefore, several key flowering homologs of *Arabidopsis* and tomato shoot apical meristem (SAM) activity genes were investigated in sweet orange by real-time PCR and *in situ* hybridization, and the results indicated that these genes were preferentially expressed in SAM as well as axillary meristem.

**Conclusion:**

Based on these findings, a model for sweet orange spring shoot self-pruning is proposed, which will enable us to better understand the mechanism of self-pruning and abscission.

**Electronic supplementary material:**

The online version of this article (doi:10.1186/1471-2164-15-892) contains supplementary material, which is available to authorized users.

## Background

Most perennial plants undergo a rhythmic periodicity for shoot growth, in which phytomers are initiated but do not fully expand, and flowering and fruiting occur only after a dormancy period [1]. Apical dominance of the terminal meristem imposes paradormancy on the lateral dormant bud, preventing them from growing out. This is termed preformation and allows for a rapid flush of growth, generally in spring [1, 2]. In shoots of many adult woody perennials, growth cessation occurs soon after this time and is followed by the abortion of the spring shoot such as citrus, kiwi fruit, peach and pomegranate. Abortion of spring shoot or abortion of spring shoot tip is an inherent characteristic that induces subsequent development from subjacent axillary buds, resulting in the sympodial growth pattern [3]. Sympodial branching is a common feature of many woody trees and a process of shoot tip abortion and pseudoterminal renewal branching from an axillary bud. In addition, shoot tip abortion plays an important role in floral bud initiation of some important fruit crops [3–5]. Although the phenomenon of shoot tip abortion is described in the older botanical literature and the resultant occurrence of a “pseudoterminal bud” is commonly used as a distinguishing characteristic in taxonomic keys to woody plants, little regarding its morphogenetic aspects has been reported until recently [3].

In citrus, there are three important types of shoots produced during the growing season. The main type grows in late winter or early spring (spring shoots), and two additional types grow at the end of June (summer shoots) and late in September (autumn shoots) [5]. The spring flush is the most important for flower formation and flowering [4, 5]. In most cases, only vegetative shoots are formed in the summer and autumn. All three types of citrus shoots typically cease growth temporarily by abortion of the shoot tips (0.5-2 cm). For spring shoots of trifoliate orange (*Poncirus trifoliata* L. Raf.), abortion of the shoot tips (0.5-1 cm) takes place in spring or early summer and is rapidly followed by a decline in the growth of the distal portion of the extending shoot. The entire shoot tip soon turns yellow and abscises at the base of the shoot apex; this physiological phenomenon is called “self-pruning” in citrus. Self-pruning is a necessary but not sufficient condition for citrus flowering. Previous cytological studies revealed that the floral buds of spring shoots in an early-flowering mutant of trifoliate orange (precocious trifoliate orange) initiated differentiation immediately after self-pruning [4]. In sweet orange, the new terminal bud and lateral buds of the spring shoot are in an undetermined state after self-pruning, and floral primordial are not observed. Only a small portion of lateral buds developed into summer or autumn shoots in a year, and new terminal buds and remaining lateral buds of the spring shoot entered dormancy until spring of the next year. The floral buds of sweet orange initiate their differentiation on spring shoots in March of the next year. The whole integrated flower bud forms in 1.5 months and then flowering begins (unpublished data). These results suggest that self-pruning is a demarcation point for shoot apical meristem (SAM) to initiate leaf bud or floral bud development in citrus. Although self-pruning has been described as playing an important role in development process in several woody species, no satisfactory adaptive or evolutionary explanations exist for it [3].

During self-pruning of citrus, shoot tip separates from the top part of the shoots at a predetermined position (about 0.5–2 cm from the shoot tip toward the basal portion, Additional file 1: Figure S1), called the abscission zone (AZ). The cells of the AZ are small, cytoplasmically dense, and isodiametric as compared with neighboring cells, and they are responsive to signals promoting abscission [6, 7]. These signals induce enzymatic dissolution of the middle lamellae between AZ cell walls, resulting in a loss of adhesion between the organ and plant body [8]. Both external and internal factors such as fungus invasion, extreme temperatures, salinity, programmed cell death (PCD), hormone, reactive oxygen species (ROS) and water stress have been reported to be involved in organ abscission [6, 7, 9–11]. Recently developed molecular approaches have been used in abscission process in horticulture crops. In apple plants, the ABA and ethylene signaling pathways are strongly up-regulated concurrently with a specific down-regulation of gibberellin signaling in the fruits induced to abscise [12, 13]. A hypothetical model for abscission process was proposed based upon both transcriptomic and metabolic data in apple, indicating a strong link between abscission and these hormones [12]. According to this model, ABA may transiently cooperate with other hormones and secondary messengers in the generation of an intrafruit signal leading to the downstream activation of the abscission zone [12]. In addiction, previous studies have also identified transcriptional signatures associated to flower and leaf abscission in tomato [14] and citrus [15], respectively. Recently, Ludwików et al. [16] reported that the *Arabidopsis* protein phosphatase type 2C, ABI1, a negative regulator of abscisic acid signaling, was also involved in the regulation of ethylene biosynthesis under oxidative stress conditions. Meanwhile, ABI1 interacted with ACS6 and dephosphorylates its C-terminal fragment, a target of the stress-responsive mitogen-activated protein kinase, MPK6 [16]. Previously, some indirect evidence also supported a link between ROS and abscission [17]. For example, a model of stress-induced leaf abscission signalling has been already proposed [17] as well as its involvement in apple fruitlet abscission [13]. Furthermore, peroxidase activity was increased during the ethylene induced pedicel abscission in tobacco plants [18]. So far, most of the current molecular knowledge on the abscission process comes from model plants. However, there is an increasing economic interest in developing molecular approaches focused on the abscission of food and fruit crops. Therefore, investigation of the molecular events associated with self-pruning development and physiology may provide new insights into the basic biology of abscission and ultimately allow this process to be manipulated in an agriculturally favorable manner.

This study was designed to assess whether PCD is involved in citrus self-pruning and to examine the expression of genes involved in self-pruning. Therefore, self-pruning phenomena, morphology, ROS accumulation, and changes in gene expression during the self-pruning process of sweet orange were investigated. Our results from terminal deoxynucleotidyl transferase-mediated dUTP nick end labeling (TUNEL) and transmission electron microscopy (TEM) analyses suggest that PCD occurs systematically at shoot tips during the self-pruning process, and ROS-induced PCD may be involved in the process of self-pruning. In addition, we carried out a high-throughput microarray analysis of the specific gene expression occurring during self-pruning. Our results notably increase the current catalogue of genes related to the abscission process and provide new candidate genes for future biotechnological applications in citrus. To our knowledge, this is the first comprehensive report of a direct link between citrus self-pruning and PCD.

## Results

### Morphology of sweet orange spring shoots self-pruning

**Figure 1:**
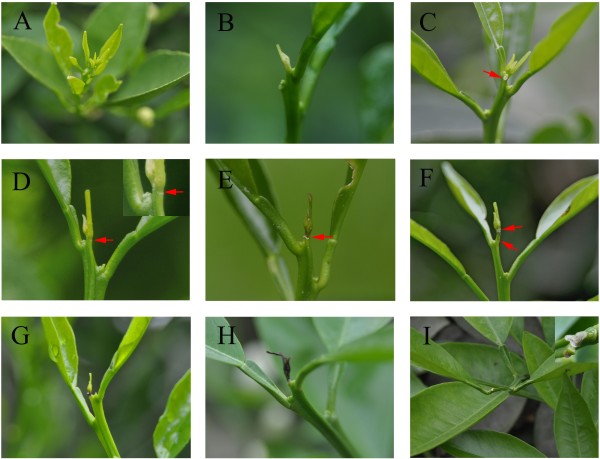
**Phenotypic characteristics of ‘Cara Cara’ navel orange (** ***Citrus sinensis*** **Osbeck) spring shoot during self-pruning process. (A)** Tips of spring shoot; **(B)** 5 days before self-pruning; **(C)** 3 days before self-pruning (lobular of shoot tips begins to fall); **(D)** begin self-pruning of spring shoots (activation AZ); **(E)** form visible AZ; **(F)** 7 days after self-pruning; **(G–H)** shoot tip gradually becomes brown and dies; **(I)** generating a protective layer for the AZ. Red arrows represent AZ.

Although sweet orange is an evergreen tree, there were no apparent developmental changes in the size or form of buds when it entered dormancy at the end of the growing season. Bud enlarge somewhat and fold back due to differential enlargement, and presumably more growth occured on the adaxial than on the abaxial surfaces in the spring. Following the period of bud opening in the current study, the shoot underwent a period of rapid elongation during which the successive leaves and the stem continued to enlarge and mature (Figure 1A). Early in this phase of growth, the tip of the shoot, including the three or four youngest pairs of leaves and leaf primordia, ceased growth (Figure 1B). This terminal part of the shoot, 4–7 mm in length, remained green for 2–3 weeks but did not increase in size (Figure 1C). About the time that the shoot attained its maximum length, the tip changed from green to yellow and then gradually became brown and died; the shoot tips on the aborting portion ranged from only 5 mm to 2 cm in length (Figure 1D–G). Yellowing occurred throughout the entire apical portion within a short time (2–3 days), and lobular of shoot tips began to fall (Figure 1C). Athough the color boundary was distinct, no depressed line commonly found in abscission layer until separation approached (Figure 1D). After such changes had begun, the AZ was evident (Figure 1E). Subsequently, an obvious area of necrosis formed across the base of the shoot tip, just above the position of the uppermost axillary buds (Figure 1 F). For spring shoots of sweet orange, self-pruning was completed within 2 weeks from lobular of shoot tips fall (Figure 1D) to generating a protective layer (Figure 1I). The shrunken, distorted shoot tips sometimes persisted for months, only gradually being sloughed off (Figure 1H).

### Cytological changes during the self-pruning process

**Figure 2:**
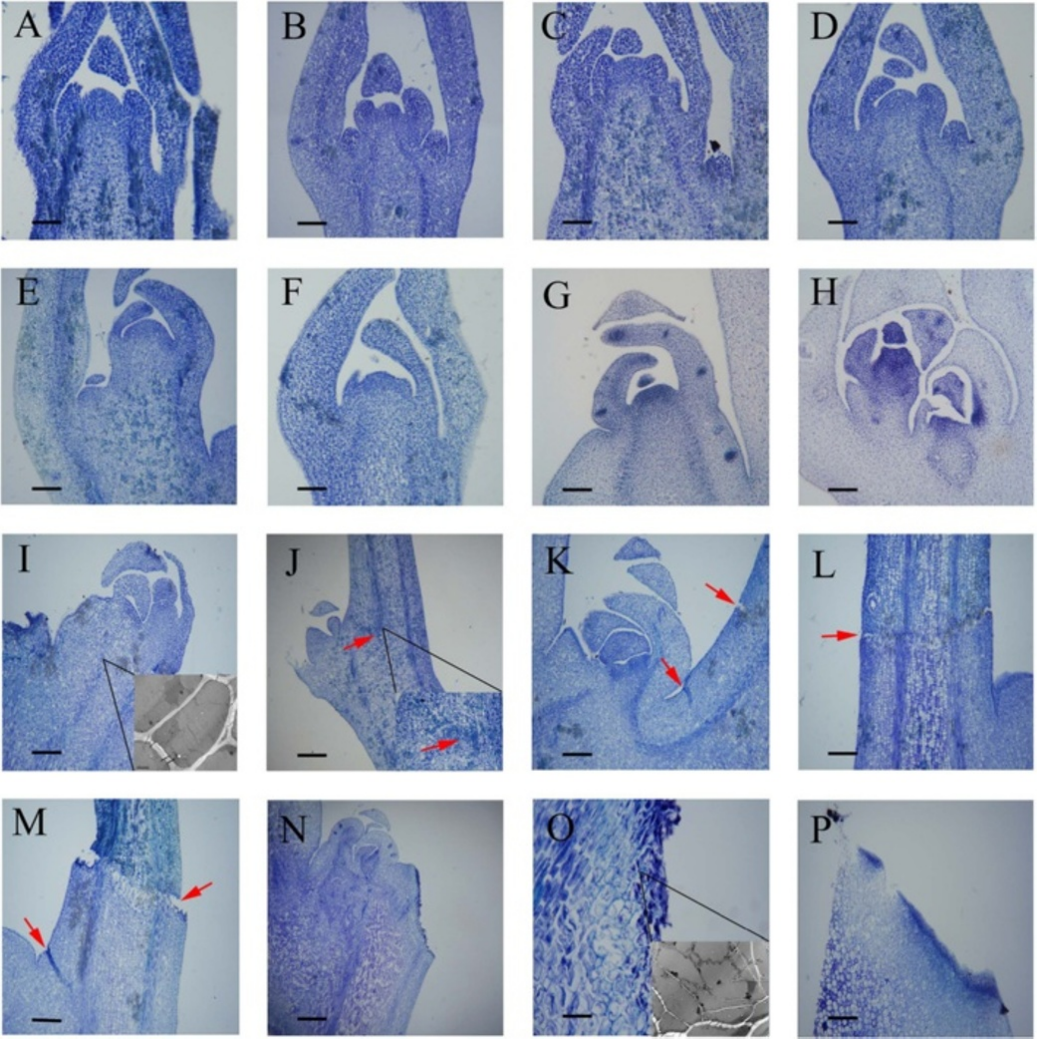
**Cytological changes of ‘Cara Cara’ navel orange (** ***Citrus sinensis*** **Osbeck) spring shoot during self-pruning process by paraffin section and TEM analysis.** Stages A–F occur before self-pruning, **(A–F)** 45 days, 35 days, 25 days, 15 days, 7 days, and 3 days before self-pruning of shoot tips, respectively. **(G)** 3 days before self-pruning of lateral bud; **(H)** 20 days after self-pruning; **(I)** lobular of shoot tips begin to fall (before self-pruning); **(J)** the appearance of the AZ; stages L–P occur after self-pruning, **(K)** visible AZ; **(L, M)** shoot tip begins to fall; **(N)** shoot tips after self-pruning; **(O)** before formation of protective layer of AZ; **(P)** after protective layer of AZ formed. Bars are 50 μm in L–P, and 100 μm in other photographs. Red arrows represent AZ.

**Figure 3:**
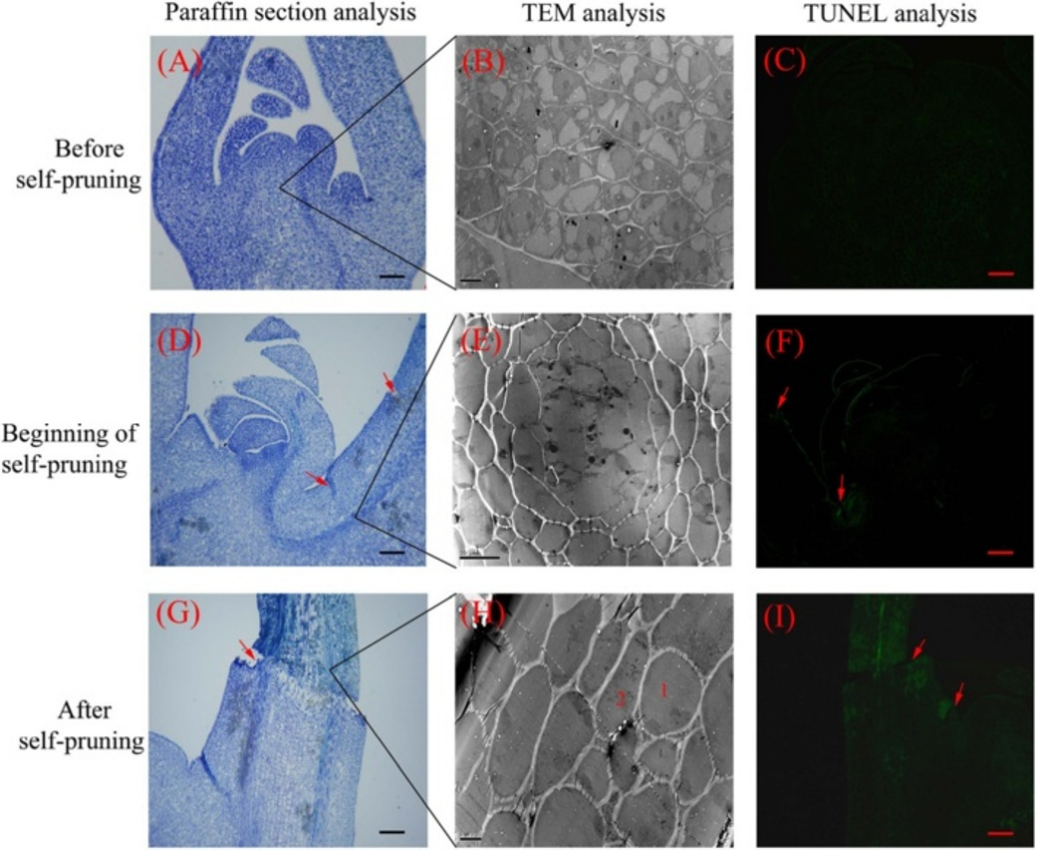
**Detection of programmed cell death in shoot tips during self-pruning process**. TUNEL analysis was performed on 10-μm-thick bud meristem sections. **(A)** Vigorous spring shoots of shoot tips (Control); **(B)** staining signal of AZ at beginning of self-pruning; **(C)** staining signal of AZ after self-pruning; **(E, F)** staining signal of the AZ at the beginning of self-pruning; **(H, I)** staining signal of AZ after self-pruning; Number 1 and 2 **(H)** represents cells undergoing programmed cell death and dying cells, respectively. Red arrows represent AZ. Paraffin section bars are 100 μm.

The paraffin sections and TEM analysis of shoot tip cells showed no visible evidence of cellular breakdown or death before self-pruning (Figures 2 and 3). Only after the shoot tip became yellow did the cytoplasm became less intensely stained compared to that in cells of the active growth regions (Figure 2A–E). This change in stainability may reflect an alteration in the chemical and/or physical nature of the protoplasm, and it was the first histological indication of an altered developmental pattern. Apices of the subjacent axillary buds would serve as the pseudoterminal bud (Figure 2G-H). In this bud, the cytoplasm became more densely stained (Figure 2H) than before self-pruning (Figure 2G). During the self-pruning process, when the shoots elongation was complete and lobular of shoot tips began to fall (Figure 2I), in the apical meristem of the unexpanded shoot tip cells became more vacuolated and their nuclei were condensed (Figures 2I and 3E). These changes suggested that a parallel senescence pattern was occurring, and scattered necrotic areas became evident in pith and cortex (Figure 2J), but at the abscission site, a separation layer in the stem was not yet apparent. After 2–3 days, the separation layer was visible (Figure 2K, L). At the later stage, when the tip including all the leaf primordia was completely necrotic, separation of the cells in pith and cortex of the stem at the abscission site had occurred (Figure 2M). The abscission site was commonly located in an internode distal to the sixth, seventh, or eighth leaf. No protective layer had formed in the stem at this stage (Figure 2N, O). After the shoot tip dropped off, the protective layer developed (Figure 2P).

### DAN degradation involved in self-pruning

To detect fragmented nuclear DNA *in situ*, the TUNEL procedure was used to assess and confirm the degradation of nuclear DNA in shoot tips (Figure 3). Vigorous growth of the shoot tips did not show symptoms of DNA fragmentation (Figure 3C). However, when self-pruning began, the initial DNA fragmentation could be detected in the AZ, and slightly more TUNEL-positive nuclei were also observed in the outer epidermis of leaf primordia (Figure 3F). As the shoot tip gradually became brown and died, widespread and more extensive DNA fragmentation was observed in the apical portions (Figure 3I). These results suggested that developmental or environmentally induced PCD occurs during the self-pruning process. The TEM results indicated that shoot tip cells are flat and small, with large nuclei and abundant cytoplasm before self-pruning (Figure 3B). These cells are rectangular with large nuclei and exhibit remarkable vitality and potential for cell division (Figure 3B). Relative to control tissues, the shoot tip cells had a markedly irregular shape after abscission induction (Figure 3E), indicating chromatin disorganization and condensation. When the AZ breakdown, the apical portions already appeared dead (Figure 3H).

### Changes in cellular and nuclear morphology of shoot tip cells by TEM analysis

**Figure 4:**
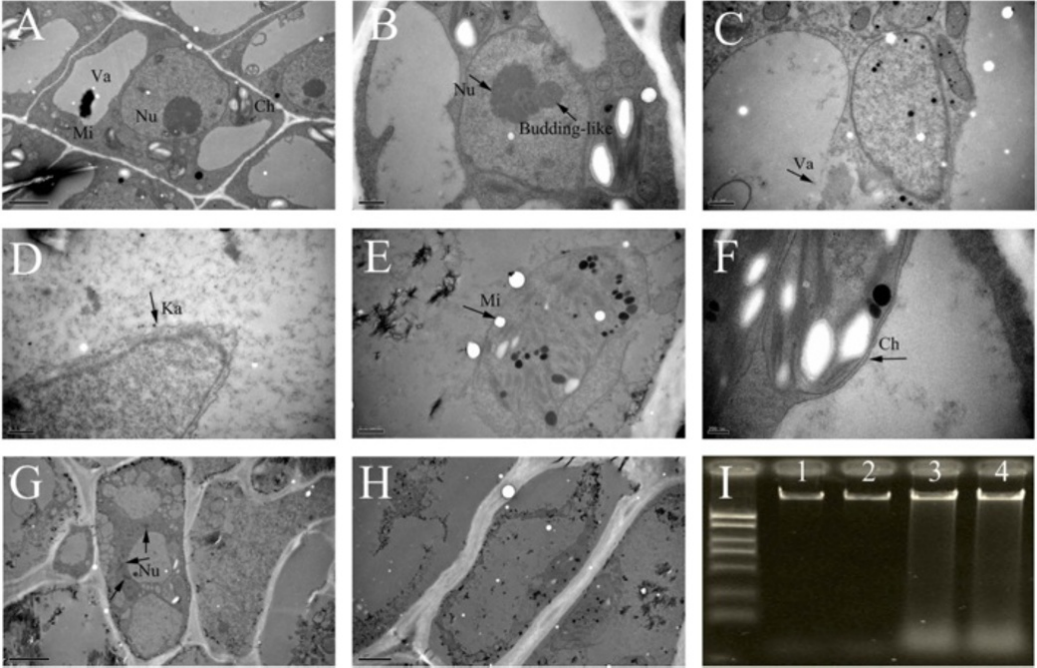
**TEM of tissue from shoot tips of spring shoots. (A)** the cell from AZ before self-pruning; **(B)** nucleolus begin death; **(C–F)** vacuole, karyotheca, mitochondria, and chloroplasts began to break, respectively; **(E)** the cells with dying bodies; **(F)** the completely dead cells; **(G)** cells with dying bodies; **(H)** the completely dead cells; **(I)** results of DNA electrophoresis of shoot tip, Dl2000 DNA molecular ladder was applied to the run, lanes 1 and 2: DNA isolated before self-pruning of shoot tip; lanes 3 and 4: DNA isolated at the start of self-pruning of the shoot tip. Cell organelle labeling: Ch, chloroplast; Va, vacuole; Mi, mitochondria; Nu, nucleus; Ka, karyotheca. Bars are 2 μm in A, G and H, 0.5 μm in C-E and 200 nm in F.

PCD in plants shows characteristic cellular, structural, and morphological features [19]. Thus, we searched for such features in the apical portion side of the AZ using TEM during the self-pruning process (Figure 4). Before self-pruning, numerous vacuoles were detected around the nucleus as well as organelles containing electron-translucent contents (Figure 4A). When self-pruning began, the cells exhibited different levels of degradation and changes that were followed by fracture development (Figure 4B-H). The cells in the AZ eventually died, and the cytoplasm appeared to be granulated (Figure 4H). Several ultrastructural changes were also detected. For example, we detected budding-like nuclear segmentations that resulted in the separation of nuclear fragments (Figure 4B, G). The tonoplast of the vacuole was ruptured and other endomembrane organelles underwent degradation (Figure 4G). Meanwhile, gradual degradation of the karyotheca, mitochondria and chloroplasts were also observed (Figure 4D-F). Overall, the observed changes in the structural and morphological features of AZ cells indicate clear differences prior to self-pruning and afterward. In addition, the DNA of apical portion cells was found to be partially degraded by using agarose-gel electrophoresis analysis as self-pruning began (Figure 4I).

### Analysis of ROS accumulation in shoot tips by histochemical staining

Histochemical staining with nitro blue tetrazolium (NBT) and diaminobenzidine (DAB) was performed to check the levels of H_2_O_2_ and O_2_^-^ of shoot tips during the self-pruning process (Additional file 2: Figure S2). The results showed similar staining patterns for both DAB and NBT. Before self-pruning, little or no staining was observed in shoot tips (Additional file 2: Figure S2G, M); whereas, the AZ was stained as self-pruning began (Additional file 2: Figure S2H, N). The shoot tips exhibited deeper staining 7 days after abscission layer formation (Additional file 2: Figure S2J, P) than 3 days after self-pruning (Additional file 2: Figure S2I, O), indicating that shoot tips accumulated higher levels of H_2_O_2_ and O_2_^-^ during the self-pruning process. The accumulation of ROS was gradually reduced after the protective layer formed (Additional file 2: Figure S2L, R).

### Differential transcriptome responses of shoot tips during self-pruning

**Figure 5:**
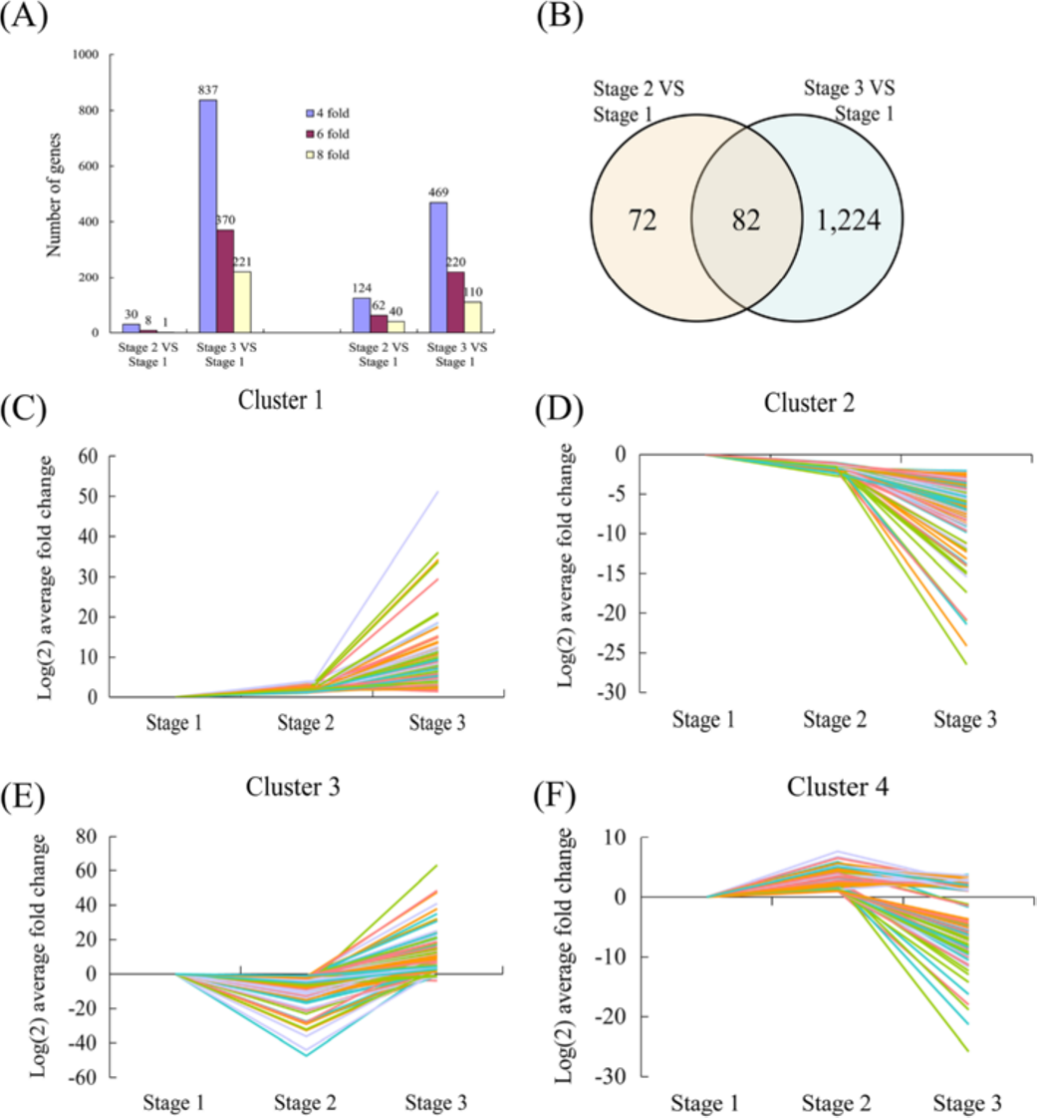
**Expression patterns of DEGs. (A)** The total numbers of DEGs (fold changes ≥ 4, 6, and 8; p ≤ 0.001) at stage 1, stage 2 and stage 3. **(B)** Venn diagram showing the overlapping of DEGs at three development stages. **(C)** Cluster 1 consisting of 571 DEGs; **(D)** Cluster 2 consisting of 337 DEGs; **(E)** Cluster 3 consisting of 335 DEGs; **(F)** Cluster 4 consisting of 135 DEGs.

To identify DEGs during the self-pruning process, a citrus microarray was used to measure the expression of genes at three stages. Among the three stages, more genes were up-regulated (15,764) than down-regulated (14,631) from stage 1 to stage 2. However, more genes were down-regulated (16,279) than up-regulated (14,116) from stage 1 to stage 3. A total of 154 DEGs were identified from stage 1 to stage 2 based on *P* ≤ 0.001 and four fold changes. Among these DEGs, 30 genes were up-regulated and 124 were down-regulated (Figure 5A). In addition, 1,306 DEGs were identified from stage 1 to stage 3, with 837 genes up-regulated and 469 down-regulated (Figure 5A). Combining the results obtained for the three stages, 1,378 DEGs were identified as candidate self-pruning-related genes; a total of 82 DEGs were in common to all three stages and may represent typical self-pruning responsive genes (Figure 5B).

In this study, we classified the 1,378 DEGs of the shoot tips into four clusters based on the similarity of the kinetic expression patterns. Cluster 1 genes (571) were induced immediately at stage 2 and most maintained high expression levels at stage 3 (Figure 5C). It was the largest group among the four clusters comprising all up-regulated genes. This cluster featured genes encoding transcription factor (TF), biotic/abiotic responses, ethylene signaling/biogenesis, and cell wall degradation enzymes. These genes were significantly induced in all three stages of self-pruning, and these results indicated that the gene cluster might play a key role during whole self-pruning process. Cluster 2 comprises 337 genes that were suppressed immediately at stage 2 and most maintained low expression levels at stage 3 (Figure 5D). BLAST analysis indicated that these genes are involved in amino acid metabolism, development and transcription, and auxin signaling. The repression of the cluster genes may imply possible involvement of meristem gene regulation and development of shoot tips. Cluster 3 comprises 335 genes that were transiently suppressed at stage 2 and were then induced at stage 3 (Figure 5E). This cluster also featured genes encoding ethylene signaling/biogenesis and cell wall degradation enzymes. This cluster show up-regulated expression at later stages of self-pruning, indicating the expression of genes involved in cell wall and ethylene metabolism. Cluster 4 comprises 135 genes that were transiently induced at stage 2 and were then suppressed at stage 3 (Figure 5F). Hormone-related genes featured this cluster, indicating these genes might be related to the response to abscission signals and the activation of the AZ cells during the abscission process.

### Identification of self-pruning–related genes by microarray analysis

To investigate the biological processes possibly regulated by the 1,378 differentially expressed genes (DEGs), a homology search was conducted using the NCBI database (Additional file 3: Table S1). We detected 1,229 sequences (89.2%) as having homology with known proteins and the remaining 149 sequences (10.8%) did not possess homology with any other proteins (Additional file 3: Table S1). In addition, 77 sequences were annotated as related to transcription factors (TFs) belonging to 13 families (Additional file 3: Table S1). The zinc finger family was the most prevalent, followed by the NAC and MYB families, part of which might play roles in regulating development and metabolism. GO annotation of these genes was also performed by Blast2GO. Based on GO annotation, only 922 DEGs (66.9%) were divided into the three principal GO organization categories: molecular function, biological process, and cellular components (Additional file 4: Figure S3). The remaining 457 DEGs (33.1%) were not classified (Additional file 3: Table S1).

Many genes involved in different hormone synthesis and signaling pathways were included among these DEGs (Additional file 5: Table S2). Four abscisic acid (ABA)-related genes (Cit.13287.1.S1_s_at, Cit.13424.1.S1_at, Cit.8654.1.S1_x_at and Cit.8661.1.S1_x_at), which encode key enzymes in ABA biosynthesis and metabolism, showed significant differences during self-pruning (two ABA 8-hydroxylase genes and two ABA stress-related proteins; Additional file 6: Figure S4). In addition, GO analysis revealed nine genes involved in ABA signaling and ABA responsiveness (Additional file 5: Table S2). The microarray results showed that most ABA-related genes were significantly up-regulated during the self-pruning process (Additional file 6: Figure S4). Twenty-four auxin-related genes were differentially altered, and these genes included six auxin-induced proteins (Cit.10311.1.S1_s_at, Cit.13997.1.S1_at, Cit.14663.1.S1_s_at, Cit.18852.1.S1_at, Cit.21592.1.S1_at and Cit.25747.1.S1_s_at), four auxin response factors (Cit.1334.1.S1_at, Cit.15798.1.S1_at, Cit.25299.1.S1_at and Cit.29400.1.S1_at), one auxin-responsive GH3 family protein (Cit.12252.1.S1_at), and 13 auxin-related genes (Additional file 5: Table S2) from GO analysis. Interestingly, the auxin-induced proteins were up-regulated and auxin response factors were down-regulated during the self-pruning process (Additional file 6: Figure S4). After self-pruning, genes for ethylene biosynthesis and perception were up-regulated (Additional file 6: Figure S4), including 16 ethylene-responsive TFs (Cit.12334.1.S1_s_at, Cit.1270.1.S1_s_at, Cit.14895.1.S1_s_at, Cit.16845.1.S1_at, Cit.17142.1.S1_s_at, Cit.19105.1.S1_at, Cit.21438.1.S1_s_at, Cit.21825.1.S1_at, Cit.22963.1.S1_x_at, Cit.2675.1.S1_s_at, Cit.29533.1.S1_s_at, Cit.3778.1.S1_at, Cit.3972.1.S1_at, Cit.4810.1.S1_at, Cit.6404.1.S1_at and Cit.6618.1.S1_at) and two ethylene response element binding proteins (Cit.24979.1.S1_at and Cit.17124.1.S1_at; Additional file 5: Table S2). This was the largest group among the five clusters comprising all hormone pathway genes. Coinciding with the increased expression of ethylene biosynthetic genes, the expression of cytokinin riboside 5-monophosphate phosphoribohydrolase gene (Cit.13613.1.S1_at), a key gene related to cytokinin biosynthesis, was consistently suppressed (Additional file 6: Figure S4). In addiction, eight genes involved in the response to gibberellin acid (GA) stimulus (Cit.11064.1.S1_at, Cit.16807.1.S1_at, Cit.19872.1.S1_s_at, Cit.26276.1.S1_at, Cit.30545.1.S1_at, Cit.35768.1.S1_s_at, Cit.36807.1.S1_s_at and Cit.6376.1.S1_at) were up-regulated after self-pruning (Additional file 5: Table S2). The expression of a GA 2-beta-dioxygenase gene, which is responsible for GA catabolism, increased. These results indicated that ABA, auxin, ethylene and GA may be involved in the regulation of self-pruning process.

In this study, a shared set of 81 genes associated with cell wall biosynthesis, loosening, and degradation were identified, with most of the genes exhibiting significant changes at all the three stages (Additional file 5: Table S2). Specifically, seven genes encoding pectinesterase-related protein (Cit.1729.1.S1_s_at, Cit.18581.1.S1_s_at, Cit.193.1.S1_s_at, Cit.28980.1.S1_s_at, Cit.29340.1.S1_s_at, Cit.31791.1.S1_at and Cit.6756.1.S1_at) and two gene encoding polygalacturonase-related protein (Cit.20071.1.S1_s_at and Cit.2559.1.S1_s_at) were up-regulated during the self-pruning process (Additional file 5: Table S2). We also observed one expansin gene (Cit.14005.1.S1_s_at) expressed during the shoot tip abscission process. Twelve genes encoding xyloglucan endotransglucosylase/hydrolase (XEHs) (Cit.10363.1.S1_s_at, Cit.1319.1.S1_s_at, Cit.1320.1.S1_s_at, Cit.15017.1.S1_at, Cit.17310.1.S1_s_at, Cit.17724.1.S1_s_at, Cit.24850.1.S1_s_at, Cit.27205.1.S1_at, Cit.30513.1.S1_x_at, Cit.5620.1.S1_s_at, Cit.9419.1.S1_x_at and Cit.9421.1.S1_s_at) were up-regulated (Additional file 7: Figure S5). Four genes encoding pectate lyase (Cit.1077.1.S1_s_at, Cit.15280.1.S1_at, Cit.3283.1.S1_s_at and Cit.35568.1.S1_s_at) were up-regulated during the whole self-pruning process (Additional file 7: Figure S5). Some candidate genes related to cell wall degradation and wall modification (Additional file 5: Table S2) were also identified, such as serine carboxypeptidase, snakin, peroxidase, cell wall invertase, and chitinase, all of which probably aid in later abscission processes (Additional file 7: Figure S5). Another group of genes that was up-regulated from stage 1 to stage 3 included those possibly involved in PCD (Additional file 5: Table S2), such as mitogen-activated protein kinase (Cit.30629.1.S1_at), beta-expansin (Cit.39752.1.S1_at), ethylene responsive element binding genes (Cit.17124.1.S1_at) and amino acid permease (Cit.18023.1.S1_at) based on GO analysis [7, 20], similar to the pattern observed for genes associated with cell wall degradation (Additional file 7: Figure S5).

### Changes in transcript levels of selected genes during the self-pruning process

Transcriptional regulation revealed by microarray data was confirmed by using real-time PCR. Twenty-four genes were chosen to design gene-specific primers; these selected genes encode proteins previously reported to be associated with, or involved in abscission process in other species, or their transcript levels were significantly changed during the whole self-pruning process. On the other hand, self-pruning plays an important role in citrus floral bud initiation. Therefore, five key or integrated citrus flowering-related genes (*APETELA1*: *CiAP1*; *FLOWERING LOCUS C*: *CiFLC*; *FLOWERING LOCUS T*: *CiFT*; and *SUPPRESSOR OF OVEREXPRESSION OF CONSTANS1/2*: *CiSOC1*) and three genes related to vegetative growth (*TERMINAL FLOWER1*: *CiTFL1*; *WUSCHEL*: *CiWUS*; and *SELF-PRUNING: CiSP*) were also investigated (Additional file 8: Figure S6). Two *SOC1-like* (*CiSOC1/2*) and three *FT* homologues from citrus were isolated in previous studies [21, 22]; however, three *CiFT* homologues showed high identities in open reading frame. Thus, total *CiFT* and *CiSOC1/2* were investigated in this study. Overall, real-time PCR revealed the same expression trend as the microarray data for 32 of the genes except Cit.14181.1 and Cit.1497.1 (Additional file 8: Figure S6), despite some quantitative differences in expression level. These results confirmed that the microarray data were reliable. It is notable that the expression levels of *CiAP1* and *CiFLC*, belonging to the cluster 1, were up-regulated whereas *CiSOC2* and *CiTFL1* were down-regulated as genes gathered in cluster 2. However, *CiSP*, *CiFT* and *CiWUS* of the cluster 3 were transiently suppressed at stage 2 and then induced to the initial expression level at stage 3 (Figure 5).

### *Expression of the CiAP1, CiFLC, CiSP and CiFT by*in situ *hybridization*

**Figure 6:**
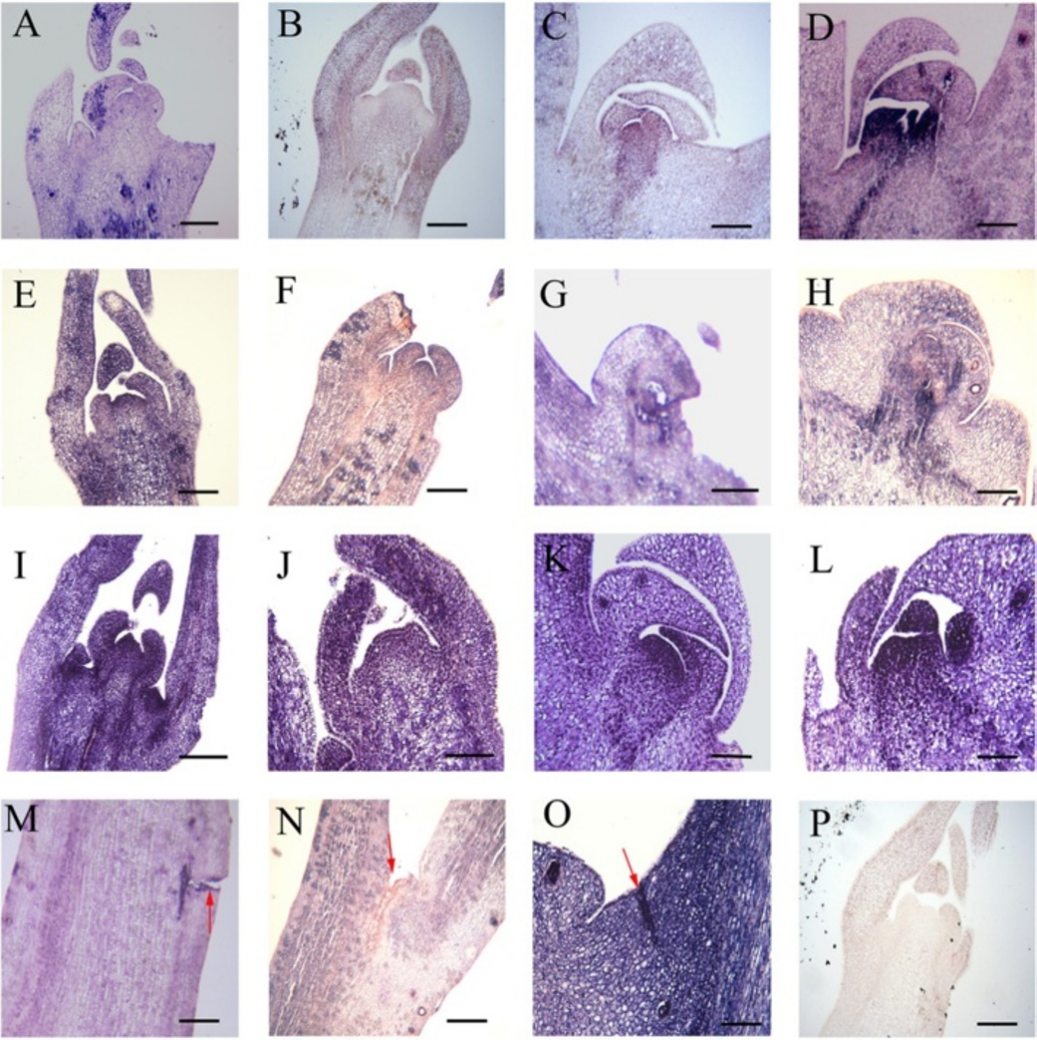
***CiAP1*** **,** ***CiFLC*** **, and** ***CiSP*** **expression during self-pruning shown by** ***in situ*** **hybridization. (A, B)** *CiAP1* expression in shoot tips at 10 days before self-pruning and self-pruning beginning, respectively; **(C, D)** *CiAP1* expression in lateral bud at 3 days before self-pruning and 20 days after self-pruning, respectively. **(E, F)** *CiFLC* expression in shoot tips at 10 days before self-pruning and self-pruning beginning, respectively; **(L, M)** *CiFLC* expression in lateral bud at 3 days before self-pruning and 20 days after self-pruning. **(I, J)** *CiSP* expression in shoot tips at 10 days before self-pruning and self-pruning beginning, respectively; **(K, L)** *CiAP1* expression in lateral bud at 3 days before self-pruning and 20 days after self-pruning, respectively; **(M, N, and O)** *CiAP1*, *CiFLC* and *CiSP* expression in AZ, respectively. **(P)** hybridized with a sense *CiSP* probe. Red arrows represent AZ. The primers used for the analyses are given in Additional file 9: Table S3. Bars are 50 μm in M, N and O, and 100 μm in other photographs.

To assess the physiological functions of *CiAP1*, *CiFLC*, *CiSP*, and *CiFT* during the self-pruning of shoot tips and lateral bud development process, we examined their expression in shoot tips and lateral bud by *in situ* hybridization (Figure 6).Primer sequences were shown in detail in the Additional file 9: Table S3. Previous studies revealed that AP1 can be used as a good marker to determine whether herbaceous and woody plants are either at the flowering stage [23, 24] or at the development stage [25]. In the present study, there was little or no *CiAP1* expression in the center of the meristem (Figure 6A, B). However, *CiAP1* was detected in leaf primordia at 10 days before self-pruning (Figure 6A) and tended to decrease at the beginning of self-pruning (Figure 6B). It is worth noting that *CiAP1* was expressed strongly in lateral buds after self-pruning (Figure 6D) as compared with its expression before self-pruning (Figure 6C). *CiFLC* is a key component in the regulatory pathway of bud dormancy release in citrus [26]. *CiFLC* was found to be expresses in the whole zone of the SAM, leaf primordia, and the young leaves at 10 days before self-pruning (Figure 6E) and its expression was maintained at low level as self-pruning began (Figure 6 F). In the lateral bud, *CiFLC* showed relatively high transcript level at 20 days after self-pruning (Figure 6H) compared with the levels at 3 days before self-pruning (Figure 6G). The level of *CiSP* was high in the whole zone of the SAM and leaf primordia at 10 days before self-pruning (Figure 6I) and tended to decrease as self-pruning began (Figure 6 J). *CiSP* was highly expressed at 20 days after self-pruning in the axillary bud (Figure 6 L) compared with 3 days before self-pruning (Figure 6 K). However, *CiSP*, *CiAP1* and *CiFLC* did not show specific expression at the AZ (Figure 6 M–O). Considering *CiFT*, we did not obtain very clear signals at these stages (data not shown), possibly because of low abundance of the transcript. In addition, recent studies of *Arabidopsis* also demonstrated that protein of *FT* is produced in the vascular tissues of leaves and moves from the leaves to the SAM as a mobile flowering signal [27].

## Discussion

Self-pruning is a demarcation point for SAM to initiate leaf bud or floral bud development. However, the phenological and morphological plasticity of self-pruning in citrus have not been examined by experimental manipulation. Abscission typically occurs at spring shoots when the citrus shoot separates from the top part of the shoots. This process has been widely characterized at AZ level [11, 14, 28, 29], as the last step involved in abscission, but understanding the mechanisms occurring during self-pruing induction would improve the sketched models already published. Therefore, this study provided the first thorough analysis of the underlying physiological and molecular activities that occur during the major events of the self-pruning process. Over the last two decades, genes related to fruit and leaf abscission have been identified, including several transcription factor genes whose homologs are involved in meristem cell fates of model plants, including *LATERAL ORGAN BOUNDARIES DOMAIN PROTEIN 1*, *WUS*, *KNAT6*, *BELL-like protein 1*, and *JOINTLESS* as well as axillary meristem genes *BLIND* and *LATERAL SUPPRESSOR* in tomato [30–34]. Although the complete sweet orange genome sequence is now available [35], no clear homologs have yet been identified in citrus except *WUS* and *KNAT6*. It is possible that some different genes, perhaps a member of a different subfamily, perform similar functions during citrus development. In this study, homologs of *KNAT6* displayed expression profiles similar to those previously described in SAM [34], but they did not reach a significant level based on stringent value *P* ≤ 0.001 and 4-fold change. One possible reason for this observation is that the regulatory mechanism of self-pruning differs between model plants and woody plants.

Many hormones regulate the process of plant organ abscission, such as auxin, ABA, GA, jasmonic acid (JA), and ethylene, among which auxin and ethylene play important roles [7, 14]. Ethylene and ABA may play the role of “amplifiers” of the unknown signal or signals that cause all the transcriptional rearrangements observed in abscising tissues. Ethylene and ABA, in concert with secondary messengers, carry the stimuli that activate the AZ and cause the release of the tissue [12]. On the contrary, auxin and gibberellin-related genes, regulating the growing and differentiation of the tissues, are negative regulated during the abscission induction. Therefore, auxin prevents the abscission process in plants, and ethylene accelerates it [7]. As previously reported [12–14], hormones seem to play a relatively important role during the abscission process, because a majority of the transcriptionally activated genes involved in hormone signaling appear to be downstream of the induction of abscission (Additional file 6: Figure S4). In fact, the expression of many genes associated with ethylene and auxin metabolism were altered during the self-pruning process. This is consistent with the relationship between abscission and increased expression of genes for ethylene synthesis and ethylene receptors in the AZ, which has been reported in apple [36] and olive [37]. As long as the flux of auxin to the AZ is maintained, cell separation is inhibited and abscission does not happen [29]. The potential importance of auxin–ethylene crosstalk was also supported in transcriptome analysis of tomato flower AZ [14], in which auxin depletion caused altered expression of auxin-related genes in association with the acquisition of ethylene sensitivity in the flower AZ. ABA has been implicated in the regulation of stress-induced senescence [38], and it has been proposed that ABA might be correlated with the ethylene-associated abscission activation in citrus fruitlets [39]. We observed some genes involved with ABA biosynthesis and signaling and an increase in the expression of genes for ABA biosynthesis during the self-pruning process. For other hormones, the induction of rate-limiting enzyme genes for GA and JA suggests coordinated regulatory modes among these hormone-related genes, and enhanced expression of the ABA catabolism gene [40] may indicate increased ABA breakdown during the self-pruning process.

PCD is a highly organized and genetically controlled suicidal process [41]. In the developmental program of plants, legumains have been associated with the PCD of internal layers of the seed coat in *Arabidopsis* [42], with the PCD related to heat shock through a signaling pathway involving ROS and a MAP kinase in *Arabidopsis* [43], with the PCD involved in somatic embryogenesis in *Arabidopsis* [44], and with the PCD related to the release of apical dominance in potato tubers [45]. This may suggest a functional relationship between PCD and self-pruning by affecting shoot tips cell viability. Here, we detected DNA fragmentation in the shoot tips during the self-pruning process by TUNEL analysis, suggesting PCD may be involved in self-pruning process. In spring shoots, if the shoot tip is undergoing PCD, it should only be detected on the apical portions but, the TUNEL signal was detected on both sides of the AZ (Figure 3I). The result might be caused by the second self-pruning of shoot tips. For some spring shoot, it will begin the second self-pruning with great distances between AZ and pseudoterminal bud (Additional file 10: Figure S7). In addition, the AZ cells are not distinguishable from those of the adjacent tissues before activation, resulting difficult to identify the proper position of the AZ. Therefore, in the apical spring shoots including even the distal AZ the transcriptional profiling analysis was performed in this study. We know that PCD can be initiated by all types of ROS, and the ROS level is tightly regulated by the balance between production and scavenging. The shift from a signaling to a deleterious role is related to ROS exceeding a threshold level, which leads to various cellular alterations and damage [46, 47]. In the present study, we noted an increase in the H_2_O_2_ and O_2_^-^ levels in shoot tips during the self-pruning process. Although we could not decipher the mechanism of action regarding H_2_O_2_ and O_2_^-^, our work demonstrates that ROS accumulate at higher level probably because they may play a role in stimulating the expression of abscission-related genes during the induction of shoot tip abscission. On the other hand, many genes involved in ROS detoxification were also identified in this study (Additional file 3: Table S1). Therefore, we speculate a balance of preferential expression of ROS-related genes between the laminar AZ and apical portions of shoot tips during the self-pruning process. This balance would be biased toward the laminar AZ during the early events prior to detachment and to the apical portion once cell separation has started. Hence, ROS could be involved in signaling events occurring during the onset of the self-pruning process. Further research is required to establish the relationship between the occurrence of PCD and the underlying regulatory molecular mechanisms during self-pruning.

Abscission is considered to be achieved through four major steps based on a working model [11]: 1) determination of the AZ, 2) competence to respond to abscission signals, 3) activation of the abscission and 4) post abscission transdifferentiation. Thus, based on above model of abscission, at stage 1 the AZ cells may be already competent to respond to abscission stimuli at stage 1; the AZ was activated at stage 2, as consequence of the up-regulation of cell-wall degrading genes as well as defense genes, and an initial lateral breakdown of the cell layers was evident; At stage 3, the post abscission transdifferentiation where the proximal cell layers increased in volume and formed the protective layer in the present study. Therefore, the classification of the clusters into four groups suggests that the abscission process may be separated into two main phases. In the early phase, from stage 1 to stage 2 (after activation of the AZ), ethylene sensitivity and abscission competence are acquired; and in the second phase, between stage 2 and stage 3 (after shoot tip removal), the active abscission process starts and leads to shoot tip abscission. A key step in the loss of adhesion between cells within a separation layer was the induction of cell wall degrading enzymes such as polygalacturonases, which have been studied in oilseed rape and *Arabidopsis* AZ s [28, 48]. The roles of other wall-modifying proteins such as expansin, XEH, and pectinesterase have also been studied during the abscission process [49]. Previous reports have indicated that an increase in XEH, expansin, and pectate lyase correlate with organ abscission [7, 50, 51]. In the present study, numerous genes encoding above genes were found to be over-represented during self-pruning process (Additional file 5: Table S2). These results indicated that these genes may be involved in sweet orange self-pruning process. Overall, these results suggest that many genes related to cell wall degradation play an important role in regulating sweet orange self-pruning.

In this study, according to function clustering of the 1,378 DEGs by GO analysis, about 30% of the genes related to macromolecule and protein metabolism were expressed preferentially in the shoot tips during the self-pruning period (Additional file 3: Table S1). The involvement of protein biosynthesis was also supported by the induction of genes encoding translation initiation and elongation factors. This is consistent with previous reports of stimulation by protein biosynthesis within the AZ in citrus [52]. These results suggested that the specific activation of the protein metabolism within the AZ is a consequence of remodeling of protein composition coupled with the activation of hormone signaling events. In the three stages of our experimental set-up, it is noteworthy that some of the crucial genes taking part in cell wall remodeling already exhibited high or low expression levels, especially in the third period. This indicated that several steps need to occur from self-pruning–related gene expression to shoot tip abscission, including protein translation and degradation and probably transport to the extracellular matrix. Indeed, our results also identified the induction of several genes (vacuolar protein sorting associated protein, GDSL esterase/lipase, and polyubiquitin) involved in vesicle trafficking based on GO analysis (Additional file 3: Table S1), a process that has recently been indicated as crucial for abscission [53]. In general, engaged lipid metabolism usually involves an extensive network of Golgi bodies and endoplasmic reticulum [54], a characteristic of cells undergoing abscission [55]. Along the same lines, the lipid metabolism (e.g., glycerolipid, glycerophospholipid, steroid, fatty acid, sphingolipid) pathway displayed a high frequency of preferential expression within the AZ (Additional file 4: Figure S3B; Additional file 3: Table S1). Therefore, the lipid metabolism pathway enhancement during abscission could be due partially to the production of Golgi bodies and new endoplasmic reticulum profiles that are generated to assist the required membrane trafficking.

In addition, self-pruning in citrus affects the SAM development program and therefore the architecture of the plant as well as the production of fruits. The floral bud induction could be paralleled with the mechanism occurring in the other fruit plants promoting the return of the bloom or the onset of alternative bearing, this latter being an unwanted trait that negatively affects fruit production. Several classes of TFs exhibited significant changes in expression, including ERF/AP2 TFs, bZIP proteins, MADS-box and MYB domain proteins (Additional file 3: Table S1). The differentially expressed ERF/AP2 TFs were co-expressed with the genes for biosynthesis and signaling of ethylene and ABA, consistent with their roles in these two hormone signaling pathways [12, 25, 52, 56]. Interestingly, three homologs of citrus flowering related genes, which encode three MADS-box TFs (*CiAP1*, *CiFLC* and *CiSP*) and regulator of self-pruning process of sweet orange spring shoots, were down-regulated in shoot tips before self-pruning, and up-regulated in lateral buds after self-pruning. Based on these results, we conclude that a high expression level of three flowering time genes might help to maintain the terminal buds in a dormant state before self-pruning, whereas the down-regulation of these genes after self-pruning induction might be closely related to the shift of cell activity or the change in flowering competence of spring shoot lateral buds.

## Conclusion

**Figure 7:**
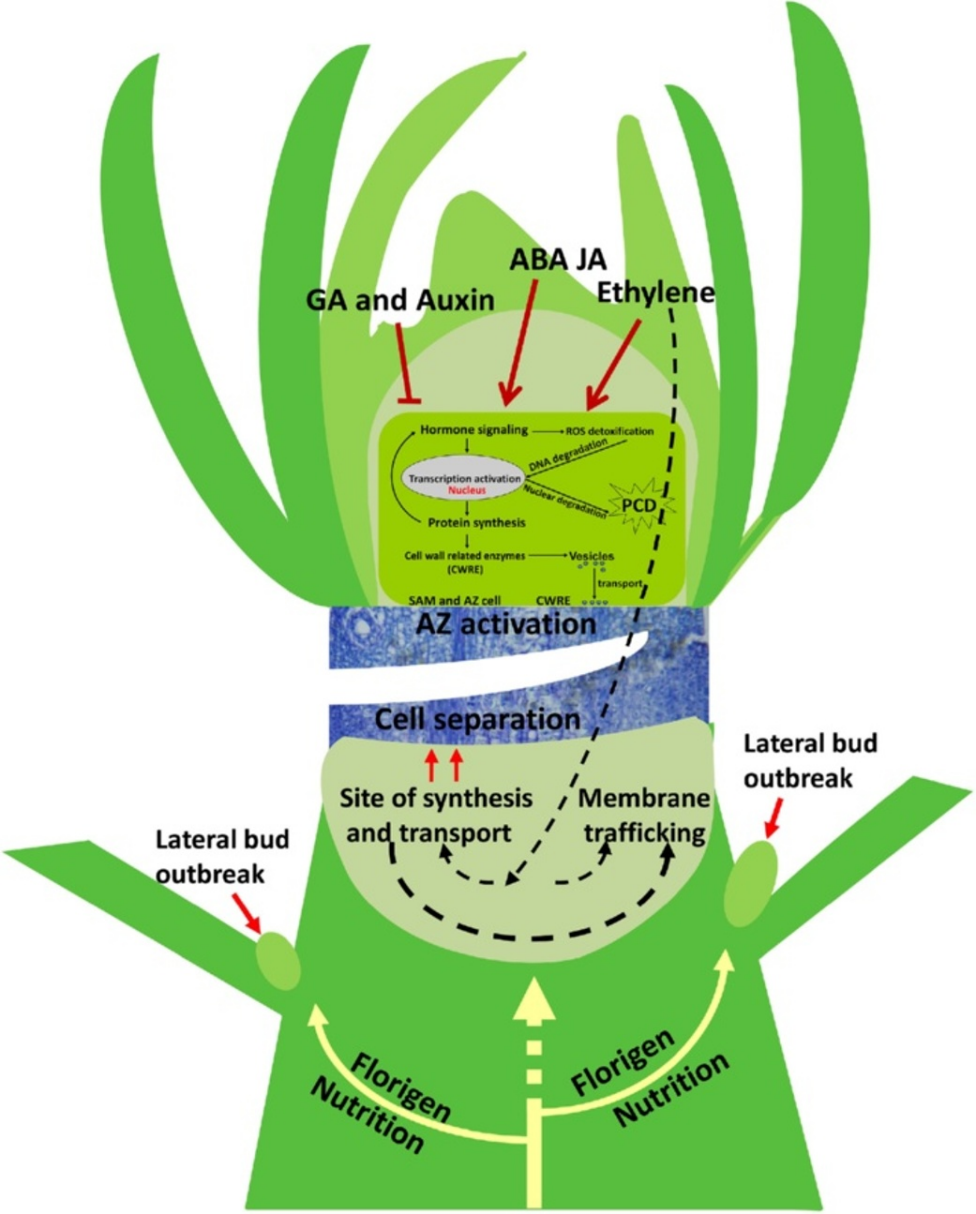
**Proposed model for molecular events occurring in the sweet orange (** ***Citrus sinensis*** **) shoot tips and AZ during self-pruning based on expression data obtained from microarray hybridization.** Arrow-ended and blunt-ended lines represent process induction and repression, respectively; white dashed and solid arrows represent transport gradually decreased and increased, respectively.

To identify the physiological and molecular properties of citrus shoot tips during the self-pruning process, we analyzed morphology, cytology, DNA degradation, ROS accumulation, and gene expression profiles of shoot tips. Based on our findings, we have developed a model of self-pruning (Figure 7). Before self-pruning, ethylene and ABA are produced in the shoot tip, triggering those unidentified early abscission signals that at the end stimulate the expression of genes involved in the cell wall metabolism. The depletion of the auxin in the AZ of the spring shoot causes the AZ to become sensitive to ethylene and ABA, which promote the advancement of abscission. The generation of Golgi-derived vesicles containing cell–wall related enzymes is responsible for the transport of these enzymes to the extracellular matrix, facilitating degradation of cell wall of the AZ cells. PCD is induced at the distal side of the AZ by ROS. The AZ is identified by loss of cell viability, altered nuclear morphology, DNA fragmentation, elevated levels of ROS, and elevated enzymatic activities and expression of PCD-associated genes (Figure 7). Throughout the entire process, the protein metabolism machinery appears to be activated to coordinate new protein scenarios, and hormone signaling and ROS are activated to regulate the steps of the process. After self-pruning, when the lateral buds are released from inhibition, florigen and nutrients are gradually transported to the lateral bud, and the lateral bud begins to accumulate nutrients for flower bud differentiation.

## Methods

### Plant materials and total RNA extraction

Plants of sweet orange (*Citrus sinensis* Osbeck ‘Cara Cara’, navel orange) were grown under natural environmental conditions in experimental fields of the National Citrus Breeding Center of Huazhong Agricultural University, Wuhan, China (30°28′ N, 114°21′ E, 30 m a.s.l.). The shoot tips of spring shoots were collected at three distinct phases (stage 1, 3 days before self-pruning, see Figure 1C; stage 2, beginning of self-pruning, see Figure 1D; stage 3, 7 days after self-pruning, see Figure 1F), which represent critical physiological and anatomical changes during the self-pruning process. The shoot attained its maximum length, the tip changed from green to yellow and lobular of some shoot tips begin to fall at 3 days before self-pruning; the separation layer was visible as self-pruning began; and an obvious necrosis commenced in surrounding AZ at 7 days after self-pruning. Therefore, shoot tips (about 0.5-2 cm, Additional file 1: Figure S1B) including the AZ at three stages were collected from adult trees of sweet orange, immediately frozen in liquid nitrogen, and stored at -80°C until use. Shoot samples were collected from three groups of trees (each with three trees) for replicate analysis. For morphological observation and floral development, about 600 buds or shoot tips displaying a similar growing condition were selected and tagged as they were sprouting, the self-pruning time of these shoot tips was recorded, the time span of self-pruning was analyzed based on these spring shoots self-pruning time. Twenty spring shoots were sampled every 2 days in the self-pruning stage and every 4 days thereafter, the shoot tips of these spring shoots and lateral bud of spring shoots were collected during self-pruning process, fixed, and stored in Formalin-Acetic Acid-Alcohol (FAA). Cytological observation of shoot tips and lateral buds were performed by paraffin section analysis followed the method described by Ruzin [57].

### PCD markers, O^2–^ and H_2_O_2_ detection

For TUNEL staining, fixed tissues were rehydrated with Histoclear and decreasing concentrations of ethanol (100%, 70%, and 30%). Tissue permeabilization was performed with 20 μg/mL proteinase K (Invitrogen, USA) in 10 mM Tris (pH 7.5) and 5 mM EDTA (pH 8) at 37°C for 30 min. After washing the tissue twice with phosphate-buffered saline (PBS), lysing enzyme (4 mg/mL) in 5 mM EDTA (pH 8) was added and incubated for 20 min at 37°C. TUNEL reaction was performed on slides using the DeadEnd Fluorometric TUNEL System (Promega, USA) according to the manufacturer’s instructions.

Accumulation of O_2_^-^ and H_2_O_2_ was detected by a histochemical staining method by using NBT and DAB, respectively [58].

### TEM analysis

The shoot tips of spring shoot were washed with PBS (pH 7.2) at room temperature and post-fixed in 2% (w/v) OsO_4_ in PBS (pH 7.2) for 3 h. The tissues were then rinsed twice in PBS and stained with uranyl acetate. The samples were dehydrated by passing them through an ethanol series and acetone, and they were then embedded in Agar100 epoxy resin (Agar Scientific). Thin sections were cut, treated with uranyl acetate/lead citrate, and examined with a Tecnai G2 Spirit transmission electron microscope (FEI; Phillips). Representative photographs are presented.

### DNA extraction and analysis

Genomic DNA from the shoot tips of spring shoot was isolated using cethyltrimethylammonium bromide (CTAB) method [59]. DNA quantity and quality were assessed spectrophotometrically at 260, 280 and 230 nm. About 5 μg of DNA was separated on 1.5% agarose gel, stained with ethidium bromide, and visualized using a UV transilluminator (Bio-Rad) by image analysis, using the Bio-Rad image analysis program.

### RNA isolation, microarray hybridization and functional annotations of the DEGs

Three total RNA samples (stage 1, stage 2, stage 3) from shoot tips including AZ were independently isolated from each sample, according to a previous protocol [60]. Hybridized with commercial Genechip Citrus Genome Arrays (Cat. no. 900732; Affymetrix; Santa Clara, CA, USA), which contains 30,171 probe sets representing 33,879 citrus transcripts. The array is based on expressed sequence tags obtained from several *Citrus* species and hybrids. Also included are sequences from Poncirus species and *Poncirus* × *Citrus* hybrids. Hybridization signals were normalized using the Affymetrix Microarray Suite program (version 5.0) and visualized using the software tool of The Institute for Genomic Research (TIGR) MeV [61]. Affymetrix raw data files (cell intensity [CEL] files) were first analyzed with robust multi-array Average (RMA) normalization as implemented in the Affymetrix Expression Console Software (version 1.1) to remove between-array effects and to standardize the low-level data [62]. In order to detect DEGs, Significance Analysis of Microarrays (SAM) algorithm [63] was used to calculate the p-values for genes at the indicated time points. The list of DEGs at each indicated time point was obtained by SAM with the fold change ≥ 4 and *P* ≤ 0.001 compared with the control.

Annotations of putative functions for DEGs were performed using the program Blast2GO [64], which was run locally to perform a BLAST search against a reference database that stores UniProt entries and their associated Gene Ontology (GO) Slim. The GO categorization results were expressed as three independent hierarchies pertaining to biological processes, cellular components, and molecular functions.

### Real-time quantitative PCR

The transcriptional profiles of 32 genes were analyzed by real-time PCR using the SYBR Green PCR master mix (Roche Applied Science, Mannheim, Germany), as described previously [26]. Primer sequences were shown in detail in the Additional file 9: Table S3. Three biologic replicates and four technical replicates were assayed, and all showed similar trends. Data from one biologic repeat are presented.

### RNA in situ hybridization and detection

Digoxigenin-labeled RNA probes were prepared using a DIG Northern Starter Kit (Roche, Germany). T7 and SP6 RNA polymerase were used to generate the sense and antisense RNA probes by *in vitro* transcription according to the manufacturer’s instructions. Prehybridization, hybridization, washing, and detection were performed as described in the Cold Spring Harbor *Arabidopsis* Molecular Genetics Course (http://www.Arabidopsis.org/cshl-course/5-in_situ.html).

#### Data access

The microarray data have been submitted to Gene Expression Omnibus (GEO) under accession no. GSE53579.

## Electronic supplementary material

Additional file 1: Figure S1: Spring shoot of sweet orange regions used in this study: (A) shoot tip regions; (B) microarray analysis regions; (C) AZ; (D) apical portions; and (E) basal portions. (DOC 1 MB)

Additional file 2: Figure S2: Histochemical staining assay of ROS accumulation with nitro blue tetrazolium (NBT) and diaminobenzidine (DAB) in shoot tips during self-pruning process. (A–F) control: shoot tips subjected to dehydration by alcohol. (G–L) NBT staining of shoot tips during self-pruning process. (M–R) DAB staining of shoot tips during self-pruning process. (A, G, and M) 10 days before self-pruning; (B, H, and N) beginning of self-pruning of spring shoots; (C, I, and O) 3 days after self-pruning; (D, J, and P) 7 days after self-pruning; (E, K, and Q) before formation of protective layer of AZ; (F, L, and R) after protective layer of AZ forms. (DOC 268 KB)

Additional file 3: Table S1: Annotation of the 1,378 DEGs during self-pruning process. (XLS 370 KB)

Additional file 4: Figure S3: Characterization of 1,378 differentially expressed genes by gene ontology categories in sweet orange (*Citrus sinensis*), (A) molecular function; (B) biological process; (C) cellular component. (DOC 1 MB)

Additional file 5: Table S2: Identification of self-pruning–related genes by microarray analysis. (XLS 88 KB)

Additional file 6: Figure S4: Cluster analysis of expression profiles of hormones related DEGs at three stages. Each column represents a sample, and each row represents a single citrus transcript sequence. The bar represented the scale of relative expression levels of DEGs, and colors indicate relative signal intensities. a: these genes involved in multiple hormones metabolism and signaling; b: SA-related genes; c: Cytokinin riboside 5-monophosphate phosphoribohydrolase. TF, transcription factor. (DOC 3 MB)

Additional file 7: Figure S5: Cluster analysis of expression profiles of cell wall related DEGs at three stages. Each column represents a sample, and each row represents a single citrus transcript sequence. The bar represented the scale of relative expression levels of DEGs, and colors indicate relative signal intensities. XEH indicated Xyloglucan endotransglucosylase hydrolase. (DOC 1 MB)

Additional file 8: Figure S6: Cluster analysis of expression profiles of DEGs at three stages of sweet orange spring shoots by real-time PCR (qPCR) and microarray analysis. Each column represents a sample, and each row represents a single citrus transcript. The bar represented the scale of relative expression levels of DEGs, and colors indicate relative signal intensities. For qPCR analysis, data points represent mean ± SE of at least four replicates for the relative expression, which were normalized by the amount of the *β-actin* control expression. The primers used for the analyses are given in Additional file 9: Table S3. (DOC 4 MB)

Additional file 9: Table S3: Specific primers for real-time PCR and *in situ* hybridization. (XLS 31 KB)

Additional file 10: Figure S7: Phenotypic characteristics of ‘Cara Cara’ navel orange (*Citrus sinensis* Osbeck) spring shoot during the second self-pruning process. Red arrows represent AZ. (DOC 12 MB)
