# Supplementary material for: Involvements of PCD and changes in gene expression profile during self-pruning of spring shoots in sweet orange (Citrus sinensis)
Source: BMC Genomics. 2014 Oct 13;15(1):892. doi: 10.1186/1471-2164-15-892 (PMC4209071; doi:10.1186/1471-2164-15-892)
Supplement: Supplementary file 1 — Additional file 1: Figure S1: Spring shoot of sweet orange regions used in this study: (A) shoot tip regions; (B) microarray analysis regions; (C) AZ; (D) apical portions; and (E) basal portions. (DOC 1 MB) [file 12864_2014_6590_MOESM1_ESM.doc]

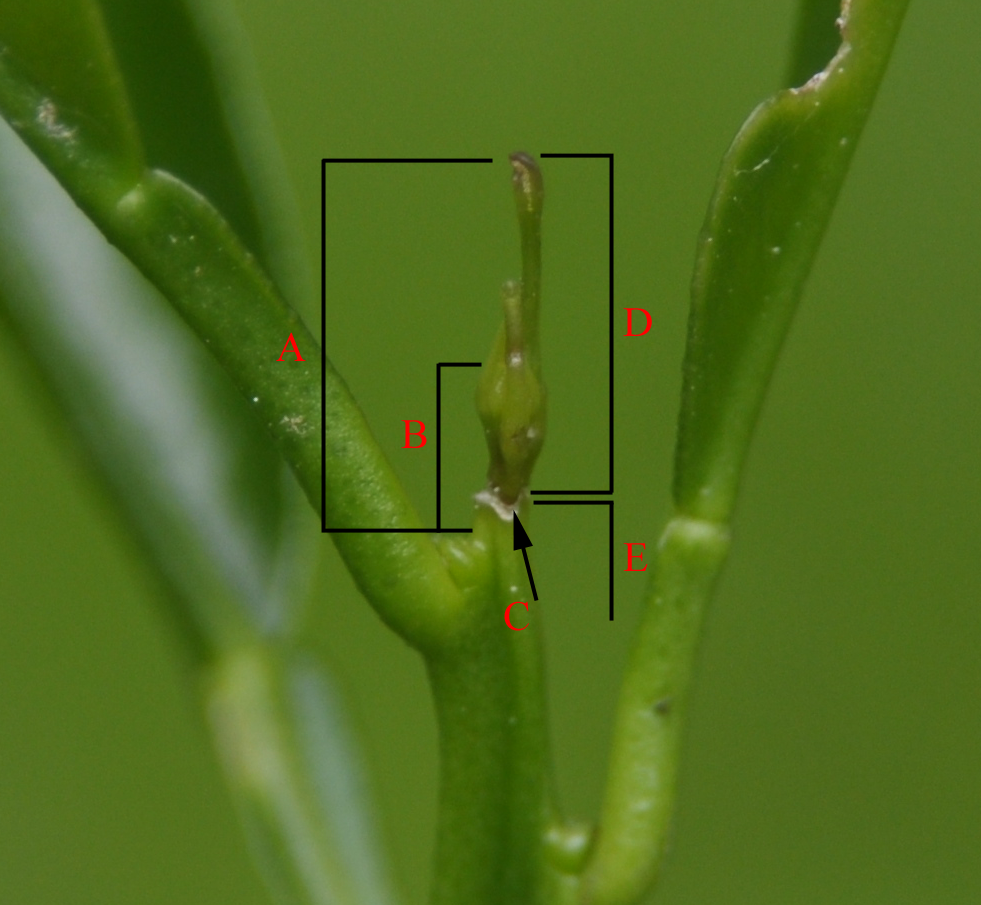


**Figure S1.** Spring shoot of sweet orange regions used in this study: (A) shoot tip regions; (B) microarray analysis regions; (C) AZ; (D) apical portions; and (E) basal portions.
