# Supplementary material for: Involvements of PCD and changes in gene expression profile during self-pruning of spring shoots in sweet orange (Citrus sinensis)
Source: BMC Genomics. 2014 Oct 13;15(1):892. doi: 10.1186/1471-2164-15-892 (PMC4209071; doi:10.1186/1471-2164-15-892)
Supplement: Supplementary file 2 — Additional file 2: Figure S2: Histochemical staining assay of ROS accumulation with nitro blue tetrazolium (NBT) and diaminobenzidine (DAB) in shoot tips during self-pruning process. (A–F) control: shoot tips subjected to dehydration by alcohol. (G–L) NBT staining of shoot tips during self-pruning process. (M–R) DAB staining of shoot tips during self-pruning process. (A, G, and M) 10 days before self-pruning; (B, H, and N) beginning of self-pruning of spring shoots; (C, I, and O) 3 days after self-pruning; (D, J, and P) 7 days after self-pruning; (E, K, and Q) before formation of protective layer of AZ; (F, L, and R) after protective layer of AZ forms. (DOC 268 KB) [file 12864_2014_6590_MOESM2_ESM.doc]

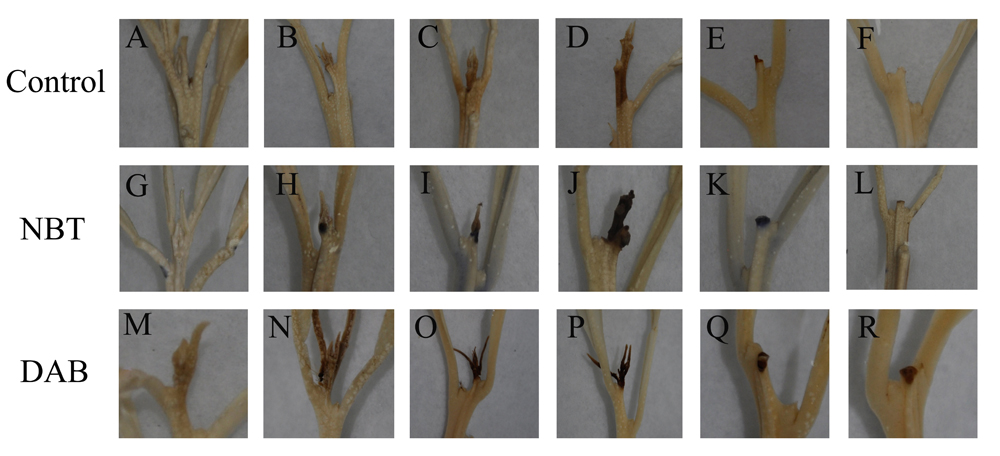


**Figure S2.** Histochemical staining assay of ROS accumulation with nitro blue tetrazolium (NBT) and diaminobenzidine (DAB) in shoot tips during self-pruning process. (A–F) control: shoot tips subjected to dehydration by alcohol. (G–L) NBT staining of shoot tips during self-pruning process. (M–R) DAB staining of shoot tips during self-pruning process. (A, C, and G) 10 days before self-pruning; (B, H, and N) beginning of self-pruning of spring shoots; (C, I, and O) 3 days after self-pruning; (D, J, and P) 7 days after self-pruning; (E, K, and Q) before formation of protective layer of AZ; (F, L, and R) after protective layer of AZ forms.
