# Supplementary material for: Involvements of PCD and changes in gene expression profile during self-pruning of spring shoots in sweet orange (Citrus sinensis)
Source: BMC Genomics. 2014 Oct 13;15(1):892. doi: 10.1186/1471-2164-15-892 (PMC4209071; doi:10.1186/1471-2164-15-892)
Supplement: Supplementary file 4 — Additional file 4: Figure S3: Characterization of 1,378 differentially expressed genes by gene ontology categories in sweet orange (Citrus sinensis), (A) molecular function; (B) biological process; (C) cellular component. (DOC 1 MB) [file 12864_2014_6590_MOESM4_ESM.doc]

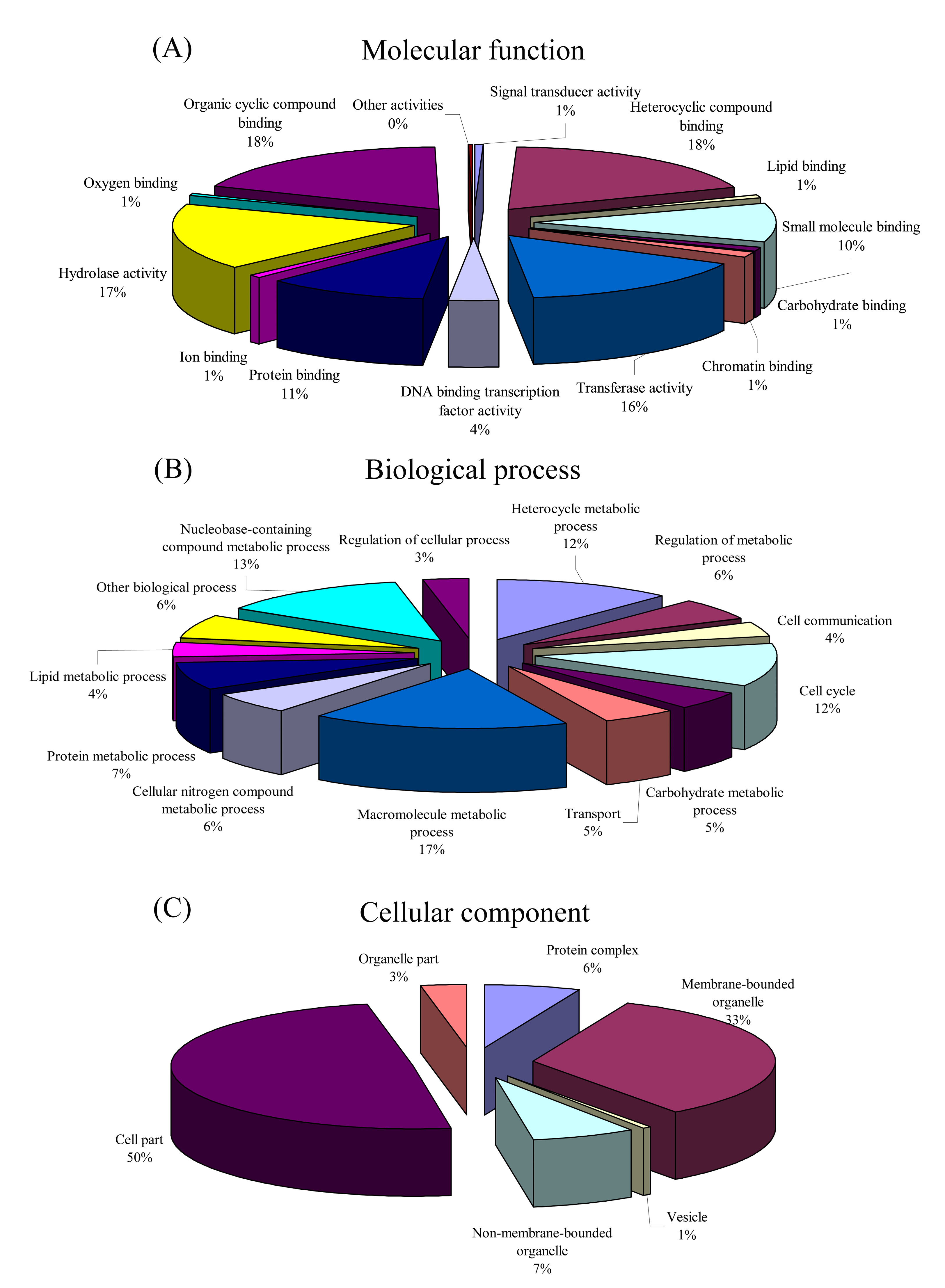


**Figure S3.** Characterization of 1,378 differentially expressed genes by gene ontology categories in sweet orange (*Citrus sinensis*), (A) molecular function; (B) biological process; (C) cellular component.
