# Supplementary material for: Involvements of PCD and changes in gene expression profile during self-pruning of spring shoots in sweet orange (Citrus sinensis)
Source: BMC Genomics. 2014 Oct 13;15(1):892. doi: 10.1186/1471-2164-15-892 (PMC4209071; doi:10.1186/1471-2164-15-892)
Supplement: Supplementary file 7 — Additional file 7: Figure S5: Cluster analysis of expression profiles of cell wall related DEGs at three stages. Each column represents a sample, and each row represents a single citrus transcript sequence. The bar represented the scale of relative expression levels of DEGs, and colors indicate relative signal intensities. XEH indicated Xyloglucan endotransglucosylase hydrolase. (DOC 1 MB) [file 12864_2014_6590_MOESM7_ESM.doc]

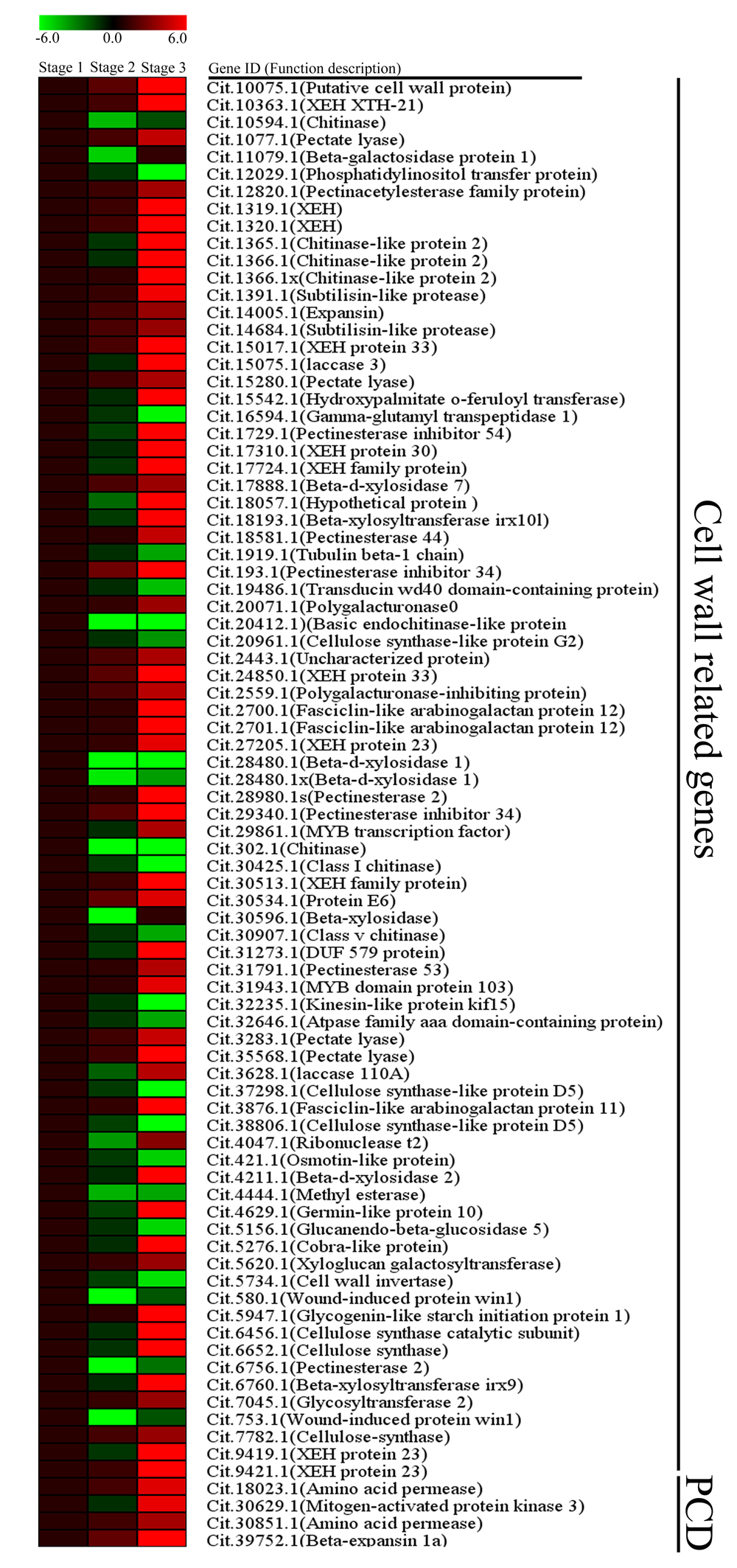


**Figure S5.** Cluster analysis of expression profiles of cell wall related DEGs at three stages. Each column represents a sample, and each row represents a single citrus transcript sequence. The bar represented the scale of relative expression levels of DEGs, and colors indicate relative signal intensities. XEH indicated Xyloglucan endotransglucosylase hydrolase
