# Supplementary material for: Involvements of PCD and changes in gene expression profile during self-pruning of spring shoots in sweet orange (Citrus sinensis)
Source: BMC Genomics. 2014 Oct 13;15(1):892. doi: 10.1186/1471-2164-15-892 (PMC4209071; doi:10.1186/1471-2164-15-892)
Supplement: Supplementary file 8 — Additional file 8: Figure S6: Cluster analysis of expression profiles of DEGs at three stages of sweet orange spring shoots by real-time PCR (qPCR) and microarray analysis. Each column represents a sample, and each row represents a single citrus transcript. The bar represented the scale of relative expression levels of DEGs, and colors indicate relative signal intensities. For qPCR analysis, data points represent mean ± SE of at least four replicates for the relative expression, which were normalized by the amount of the β-actin control expression. The primers used for the analyses are given in Additional file 9: Table S3. (DOC 4 MB) [file 12864_2014_6590_MOESM8_ESM.doc]

**Figure S6**. Cluster analysis of expression profiles of DEGs at three stages of sweet orange spring shoots by real-time PCR (qPCR) and microarray analysis. Each column represents a sample, and each row represents a single citrus transcript. The bar represented the scale of relative expression levels of DEGs, and colors indicate relative signal intensities. For qPCR analysis, data points represent mean ± SE of at least four replicates for the relative expression, which were normalized by the amount of the *β-actin* control expression. The primers used for the analyses are given in Table S3.
