# Supplementary material for: Involvements of PCD and changes in gene expression profile during self-pruning of spring shoots in sweet orange (Citrus sinensis)
Source: BMC Genomics. 2014 Oct 13;15(1):892. doi: 10.1186/1471-2164-15-892 (PMC4209071; doi:10.1186/1471-2164-15-892)
Supplement: Supplementary file 10 — Additional file 10: Figure S7: Phenotypic characteristics of ‘Cara Cara’ navel orange (Citrus sinensis Osbeck) spring shoot during the second self-pruning process. Red arrows represent AZ. (DOC 12 MB) [file 12864_2014_6590_MOESM10_ESM.doc]

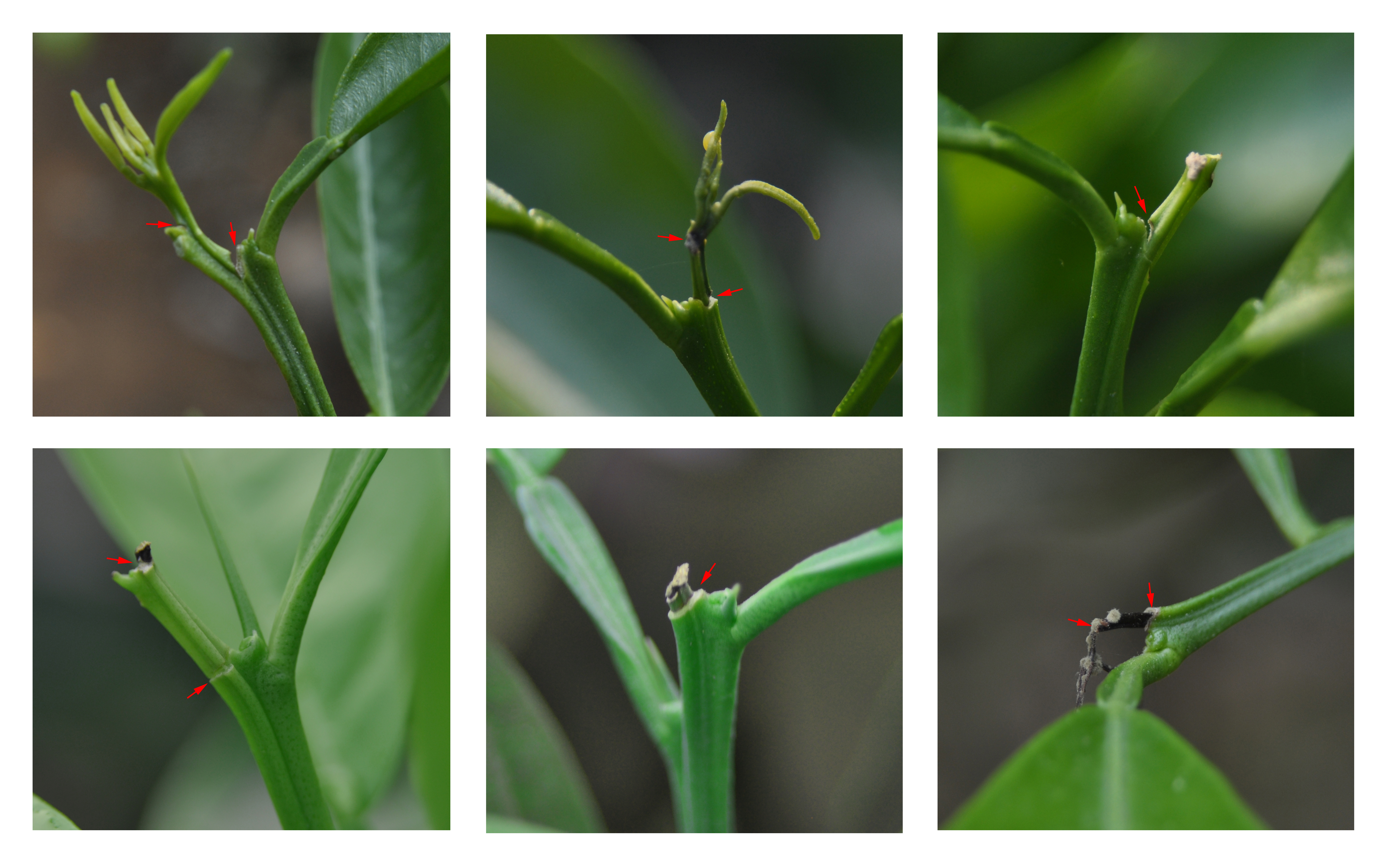


**Figure S7.** Phenotypic characteristics of ‘Cara Cara’ navel orange (*Citrus sinensis* Osbeck) spring shoot during the second self-pruning process. Red arrows represent AZ.
